# Socio-economic disadvantage and quality Antenatal Care (ANC) in Sierra Leone: Evidence from Demographic and Health Survey

**DOI:** 10.1371/journal.pone.0280061

**Published:** 2023-01-12

**Authors:** Kwamena Sekyi Dickson, Edward Kwabena Ameyaw, Mawulorm Akpeke, Barbara Elorm Mottey, Kenneth Setorwu Adde, Kobina Esia-Donkoh

**Affiliations:** 1 Department of Population and Health, College of Humanities and Legal Studies, University of Cape Coast, Cape Coast, Ghana; 2 Institute of Policy Studies and School of Graduate Studies, Lingnan University, Tuen Mun, Hong Kong; 3 Institute of Health Research, University of Health and Allied Sciences, Volta Region, Ghana; 4 Department of Environmental Health Sciences, University of Massachusetts, Amherst, Massachusetts, United States of America; Georgia Southern University, UNITED STATES

## Abstract

**Introduction:**

Reduction of maternal mortality remains a global priority as highlighted by the third Sustainable Development Goal (SDG). This is critical in the case of Sierra Leone as the country is one of three (3) countries with the highest maternal mortality ratio globally, thus 1,120 per 100,000 live births. The approximate lifetime risk of maternal mortality in the country is 1 in 17, relative to 1 in 3,300 in high-income countries. These raise doubt about the quality of the continuum of maternal healthcare in the country, particularly antenatal care and as a result, the objective of the present study is to investigate the association between socio-economic disadvantage and quality antenatal care service utilisation as well as associated correlates in Sierra Leone.

**Materials and methods:**

The study used data from the most recent Demographic and Health Survey (DHS) of Sierra Leone. Only women who had given birth in the five years preceding the survey were included, which is 6,028. Quality antenatal care was defined as receipt of recommended ANC services including uptake of recommended pregnancy drugs (e.g. Fansidar and iron supplement); injections (e.g. tetanus injection) and having some samples (e.g. blood and urine sample) and health status indicators (e.g. blood pressure) taken. An index was created from these indicators with scores ranging from 0 to 6. The scores 0 to 5 were labelled as “incomplete” and 6 was labelled as “complete” and this was used to create a dummy variable. In analysing the data, descriptive analysis was done using chi-square test as well as an inferential analysis using bivariate and multivariate models.

**Results:**

Socio-economic disadvantaged [1.46 (1.09, 1.95), place of residence [2.29 (1.43, 3.67)], frequency of listening to radio [1.58 (1.20, 2.09)], health insurance coverage [3.48 (1.40, 8.64)], getting medical help for self: permission to go [0.53(0.42, 0.69) were seen to have significant relationship with quality of ANC utilized by women during pregnancy. Also, women Mende ethnicity are more likely to utilise quality ANC compared to women from the Temne ethnicity [2.58 (1.79, 3.72)].

**Conclusion:**

Policy makers could consider measures to boost patronage of quality ANC in Sierra Leone by targeting the socio-economically disadvantaged women. Targeting these sub-groups with pro- maternal and child health (MCH) interventions would help Sierra Leone achieve Goal 3 of the SDGs.

## Introduction

Reduction in maternal mortality remains a global priority as highlighted by the third Sustainable Development Goal (SDG) [1,2]. However, it remains a global challenge because over 800 women die daily from pregnancy and childbirth-related problems [3]. Although worldwide, there has been a reduction in maternal deaths from 342 to 211 deaths per 100,000 live births between 2000 and 2017 [3] the contribution of developing countries to maternal mortality remains high with sub-Saharan Africa (SSA) accounting for about 66% of global maternal mortality cases [4]. As of 2017, South Sudan, Chad and Sierra Leone recorded maternal mortality rates (MMR) above one thousand [3]. This could be attributed to the fact that most women in SSA are socially or economically disadvantaged and therefore have a high risk of dying during pregnancy and childbirth [5]. That is, poorer women in sub-Saharan African countries like Sierra Leone lack timely access to both skilled attendants during pregnancy (antenatal care) and childbirth, unlike women in developed countries where life-saving devices and emergency procedures are readily available [5].

As such, maternal mortality has been acknowledged as a human right issue in the highest attainable standard of health [6]. This is recognised and protected by international human rights treaties such as the Convention on the Elimination of All Forms of Discrimination Against Women (CEDAW), the International Covenant on Economic, Social and Cultural Rights (ICESCR) and the laws of many countries [7,8]. Maternal mortality could be prevented with the utilization of quality antenatal care (ANC) services [9–11]. Quality ANC services promote early detection and treatment of complications thereby increasing the chances of safe delivery [12]. Despite these benefits, most pregnant women in some SSA countries do not utilize quality ANC services adequately and lose their lives at the time of pregnancy or during childbirth [13]. Distance to health facilities, limited options for transport, and a high prevalence of traditional practices and beliefs are some of the key contributors to the underutilization of quality ANC services [1,13].

Despite the regional decline in maternal mortality, Sierra Leone records the third highest cases of maternal death globally, with 1,120 deaths per 100,000 live births [14]. This is overwhelming because, with the Free Health Care Initiative (FHCI) that was introduced in Sierra Leone purposely to exempt pregnant women from paying costs associated with ANC, the country is expected to be among those with low maternal mortality rates [15]. However, the country recorded an increment of only 24% in more than four focused ANC visits within the period of 2008 to 2013 [15]. Generally, the cost of healthcare and distance to health care facilities remain barriers to accessing healthcare services (or in time) by pregnant women most especially in rural areas [16]. It is not surprising to note that maternal mortality increased during the Ebola outbreak period. Fear of contracting the disease discouraged people from visiting healthcare facilities [16,17].

Most facilities that provide delivery services, had inadequate rooms and kits for delivery and this result in poor service quality [18]. In addition, other existing literature has shown that lack of essential supplies and infrastructure at service delivery points coupled with late first ANC visits are the main barriers to the provision of ANC services in Sierra Leone [5,19]. Based on the foregoing and the paucity of the empirical literature on quality ANC, the objective of the paper is to determine the association between socio-economic disadvantage and quality antenatal care service utilisation in Sierra Leone as well as correlates of quality antenatal care. Results from this study will unearth the predictors of quality ANC utilisation in Sierra Leone. This would assist policymakers and health workers in the provision of quality ANC services for pregnant women in Sierra Leone to reduce maternal mortality and aid in achieving the first target of SDG 3.

## Materials and methods

### Data

The study used data from the most recent Demographic and Health Survey (DHS) conducted in Sierra Leone in 2019. The DHS is a country-wide representative study undertaken in a five-year period in several low- and middle-income countries (LMICs) in Asia and Africa. It focuses on maternal and child health by interviewing women in their reproductive age (15–49 years). The DHS follows a standard procedure in areas such as sampling, questionnaire, data collection, cleaning, coding, and analysis, which allows for comparability among countries. For this study, only women who have given birth in the five years preceding the surveys were included, which is 6,028.

### Definition of variables

#### Dependent variable

Table 1 presents the measurement of variables. The dependent variable is quality antenatal care (ANC) surmised from literature [20–22]. The elements of quality antenatal care consist of: 1. Took Fansidar during pregnancy; 2. Tetanus injection before birth; 3. Blood pressure taken during pregnancy; 4. Urine sample taken during pregnancy; 5. Iron supplements taken during pregnancy; and 6. Blood sample taken during pregnancy. An index was created with scores ranging from 0 to 6. The scores 0 to 5 were labeled as “incomplete” and 6 was labeled as “complete”. A dummy variable was generated with ‘0’ being women who had 0 to 5 of the elements of antenatal care and ‘1’ if women had all the six elements of antenatal care.

**Table 1:** Measurement of variables.

Variable	Measurement (code)
**Dependent variable**	
*Quality of Antenatal Care (ANC* **)**	Incomplete (ANC) = 0
	Complete (ANC) = 1
**Explanatory variables**	
*Socio-economic disadvantage*	Tertile 1 (Least disadvantage) = 1
	Tertile 2 (Moderate disadvantaged) = 2
	Tertile 3 (Most disadvantage) = 3
*Age*	
	15–19 = 1
	20–24 = 2
	25–29 = 3
	30–34 = 4
	35–39 = 5
	40–44 = 6
	45–49 = 7
*Place of residence*	
	Urban = 1
	Rural = 2
*Marital status*	
	Never in union = 1
	Married = 2
	Living with partner = 3
	Widowed = 4
	Divorced = 5
	Separated = 6
*Frequency of listening to radio*	
	Not at all = 1
	Less than once a week = 2
	At least once a week = 3
*Frequency of watching television*	
	Not at all = 1
	Less than once a week = 2
	At least once a week = 3
*Getting medical help for self*: *permission to go*	
	Big problem = 1
	Not a big problem = 2
*Getting medical help for self*: *getting money needed for treatment*	
	Big problem = 1
	Not a big problem 2
*Getting medical help for self*: *distance to health facility*	
	Big problem = 1
	Not a big problem = 2
*Health insurance coverage*	
	No = 1
	Yes = 2
*Ethnicity*	
	Creole = 1
	Fullah = 2
	Kono = 3
	Limba = 4
	Loko = 5
	Mandingo = 6
	Mende = 7
	Sherbro = 8
	Temne = 9
	Korankoh = 10
	Other
*Religion*	
	Christian = 1
	Islam = 2
	None = 3
*Region*	
	Eastern
	Northern
	North western
	Western

#### Explanatory variable and covariates

The socio-economic disadvantage variable was generated from the wealth status, education and occupation variables and captured as tertile 1(least disadvantaged), tertile 2 (moderate disadvantaged), and tertile 3 (most disadvantaged). A standardised rating was derived with an average rating (zero) and standard deviation. The rankings were then segregated into tertiles representing lower scores (tertile 1), average scores (tertile 2) and higher scores (tertile 3). Other explanatory variables or covariates include age, place of residence, marital status, frequency of listening to radio, frequency of watching television, getting medical help for self: permission to go, getting medical help for self: getting money needed for treatment, getting medical help for self: distance to health facility, health insurance coverage, ethnicity, religion, region.

### Data analysis

Descriptive and inferential analyses were conducted. The descriptive analyses (a chi-square test) focused on background characteristics and quality of ANC. Two models were used for the inferential analysis. Model 1 was a bivariate analysis that explored the relationship between socio-economic disadvantage and quality ANC. Model 2 was a multivariate analysis looked at the complete model, it controlled for the other explanatory variables. Model 2 examined the relationship between all the explanatory variables (Socio-economic disadvantage, age, place of residence, level of education, wealth status, marital status, frequency of listening to radio, frequency of watching television, getting medical help for self: permission to go, getting medical help for self: getting money needed for treatment, getting medical help for self: distance to health facility) and the outcome variable (Quality of Antenatal Care (ANC**)**.

Specifically, Binary Logistic Regression was conducted. A key assumption underlying the binary logistic regression model is that the dependent variable should be dichotomous in nature and the data should not have any outlier. The formulae behind the binary logistic regression is given as;

Let Y be a dichotomous variable which is defined as

$$Y=\left\{ \begin{array}{c} 1\;for\;the\;women\;who\;had\;all\;the\;six\;elements\;of\;antenatal\;care\\ 0\;for\;women\;who\;had\;0\;to\;5\;of\;the\;elements\;of\;antenatal\;care \end{array}\right.$$

and p = Pr (Y = 1|X_1_,…,X_k_).


$$p=\frac{1}{1+\exp[-(\beta_{0}+\beta_{1}X_{1}+\beta_{2}X_{2}+\ldots +\beta_{k}X_{k})]}$$

$$\mathrm{and}\;\hat{p}=\frac{1}{1+\exp[-({\hat{\beta }}_{0}+{\hat{\beta }}_{1}X_{1}+{\hat{\beta }}_{2}X_{2}+\ldots +{\hat{\beta }}_{k}X_{k})]}$$

Note: With no predictors, $\hat{p}=\frac{\sum_{i=1}^{n}Y_{i}}{n}=\bar{Y}$

The model was used to examine the relationships between the explanatory factors and the outcome variable. All results of the binary logistic analyses were presented as odds ratios (ORs) and adjusted odds ratios (AORs) with 95% confidence intervals (CIs). Normative groups were chosen as reference categories in the models [23]. The *svy command* was used to account for the complexity of the sampling of the DHS. All analyses were done using Stata version 14.

### Ethical approval

This study benefited from a set of publicly available data from DHS. The DHS Program adheres to ethical standards to protect respondents’ privacy. Inner-City Fund (ICF) International and the National Institute of Statistics (Institut Nationale de la Statistique) also ensure that the DHS surveys comply with the ethical requirements of Health and Human Services. For this study, no additional ethical approval was required because the data is secondary and publicly available. Details of the ethical standards are available at http://goo.gl/ny8T6X.

## Results

### Background characteristics and quality antenatal care

Table 2 presents the background characteristics and proportion of quality antenatal care received. Moderately disadvantaged women (91.1%), women aged 20–24 (89.8%) as well as and urban residents (93.5%) dominated in accessing quality ANC. Similarly, women who had never been in union (91.3%) dominated in access to quality ANC. It was also evident that 92.6% of women who listened to radio at least once a week, and 94% of those who watched television, at least, once a week had quality ANC just as the proportion of women who admitted that getting permission to access healthcare was not a big problem (90.7%). Additionally, receipt of quality ANC dominated among women who indicated that getting money needed for treatment (89.5%) and distance to health facility were not big problems (90.1%). Similarly, those with health insurance coverage (97.7%), from other foreign ethnicity (100%), identified as Islam (88.5%) and from the Western region (95.2%) had a higher proportion of women receiving quality ANC.

**Table 2:** Background characteristics and proportion of quality antenatal care received.

Background characteristics	Frequency (n = 6,028)	Percentage (%)	Proportion of women who had quality ANC	Chi Square (P value)
**Socio-economic disadvantage**				94 (0.000)
Tertile 1 (Least disadvantage)	1,626	27.0	90.9	
Tertile 2 (Moderate disadvantaged)	2,149	35.6	91.1	
Tertile 3 (Most disadvantage)	2,253	37.4	82.9	
**Age**				14 (0.025)
15–19	505	8.4	88.2	
20–24	1,328	22.0	89.8	
25–29	1,565	26.0	87.8	
30–34	1,055	17.5	87.4	
35–39	1,024	17.0	88.4	
40–44	369	6.1	82.7	
45–49	182	3.0	87.0	
**Place of residence**				81 (0.000)
Urban	2,111	35.0	93.5	
Rural	3,917	65.0	85.0	
**Marital status**				11 (0.043)
Never in union	823	13.6	91.3	
Married	4,693	77.9	87.6	
Living with partner	276	4.6	86.4	
Widowed	90	1.5	84.3	
Divorced	13	0.2	86.1	
Separated	133	2.2	87.3	
**Frequency of listening to radio**				47 (0.000)
Not at all	3,492	57.9	85.9	
Less than once a week	1,069	17.7	88.3	
At least once a week	1,467	24.4	92.6	
**Frequency of watching television**				31 (0.000)
Not at all	4,710	78.1	86.8	
Less than once a week	623	10.3	90.1	
At least once a week	695	11.6	94.0	
**Getting medical help for self: getting permission to go**				125 (0.000)
Big problem	1,514	25.1	79.9	
Not a big problem	4,514	74.9	90.7	
**Getting medical help for self: getting money needed for treatment**				7 (0.011)
Big problem	4,421	73.3	87.4	
Not a big problem	1,607	26.7	89.5	
**Getting medical help for self: distance to health facility**				31 (0.000)
Big problem	2,961	49.1	85.8	
Not a big problem	3,067	50.9	90.1	
**Health insurance coverage**				24 (0.000)
No	5,762	95.6	87.5	
Yes	266	4.4	97.7	
**Ethnicity**				188 (0.000)
Creole	34	0.6	91.3	
Fullah	166	2.8	91.4	
Kono	216	3.6	64.3	
Limba	503	8.3	92.8	
Loko	104	1.7	91.7	
Mandingo	126	2.1	90.9	
Mende	2003	33.3	87.2	
Sherbro	131	2.2	68.3	
Temne	2,178	36.1	90.7	
Korankoh	267	4.4	87.6	
Other	300	4.9	86.6	
**Religion**				7 (0.022)
Christian	1,253	20.8	85.9	
Islam	4,773	79.1	88.5	
None	2	0.1	65.2	
**Region**				171 (0.000)
Eastern	1,292	21.4	81.6	
Northern	1,324	22.0	91.7	
North western	1,289	21.4	91.6	
Southern	1,186	19.7	81.0	
Western	937	15.5	95.2	
Total	6,028	100	87.9	

Source: Computed from the 2019 Sierra Leone Demographic and Health Survey.

#### Inferential results

As illustrated in Table 3, moderate disadvantaged women had higher odds of receiving quality ANC relative to most disadvantaged women (aOR = 1.46, 95% CI = 1.09, 1.94). Compared with urban residents, women in rural locations had higher odds of receiving quality ANC (aOR = 2.29; 95% CI = 1.43, 3.67). Higher odds of quality ANC were also noted among women who reported that they listened to radio, at least, once a week (aOR = 1.58; 95% CI = 1.20, 2.09) (see Table 3). Meanwhile, women who indicated that getting permission to seek medical care was a big problem had lower odds of ANC (aOR = 0.53; 95% CI = 0.42, 0.69) compared to those who reported that getting permission was not a big problem.

**Table 3:** Binary logistic regression results on explanatory variables and quality ANC.

Explanatory variables	Model 1	Model 2
	Odds Ratio (Confidence Interval)	Adjusted Odds Ratio (Confidence Interval)
**Socio-economic disadvantaged**		
Tertile 1 (Least disadvantaged)	2.06***(1.59, 2.52)	0.90(0.51, 1.56)
Tertile 2 (Moderate disadvantaged)	2.12***(1.77, 2.53)	1.46***(1.09, 1.95)
Tertile 3 (Most disadvantaged)	Ref	Ref
**Age**		
15–19		1.03(0.74, 1.43)
20–24		1.14(0.87, 1.49)
25–29		Ref
30–34		1.10(0.84, 1.43)
35–39		1.22(0.92, 1.62)
40–44		0.79(0.56, 1.11)
45–49		1.34(0.82, 2.20)
**Place of residence**		
Urban		Ref
Rural		2.29***(1.43, 3.67)
**Marital status**		
Never in union		1.36(0.98, 1.89)
Married		Ref
Living with partner		0.94(0.65, 1.35)
Widowed		0.76(0.30, 1.92)
Divorced		0.78(0.14, 4.39)
Separated		0.94(0.55, 1.61)
**Frequency of listening to radio**		
Not at all		Ref
Less than once a week		1.11(0.85, 1.45)
At least once a week		1.58***(1.20, 2.09)
**Frequency of watching television**		
Not at all		Ref
Less than once a week		0.99(0.68, 1.45)
At least once a week		0.87(0.53, 1.44)
**Getting medical help for self: getting permission to go**		
Big problem		0.53***(0.42, 0.69)
Not a big problem		Ref
**Getting medical help for self: getting money needed for treatment**		
Big problem		Ref
Not a big problem		0.80(0.61, 1.06)
**Getting medical help for self: distance to health facility**		
Big problem		1.11(0.88, 1.40)
Not a big problem		Ref
**Health insurance coverage**		
No		Ref
Yes		3.48**(1.40, 8.64)
**Ethnicity**		
Creole		0.84(0.13, 5.28)
Fullah		1.28(0.67, 2.43)
Kono		0.69(0.40, 1.19)
Limba		1.37(0.89, 2.12)
Loko		1.25(0.46, 3.37)
Mandingo		1.25(0.63, 2.37)
Mende		2.58***(1.79, 3.72)
Sherbro		0.79(0.44, 1.42)
Temne		Ref
Korankoh		0.95(0.74, 2.17)
Other		1.32(0.77, 2.27)
**Religion**		
Christian		Ref
Islam		1.21(0.98, 1.50)
None		0.17(0.16, 1.78)
**Region**		
Eastern		0.29***(0.18, 0.46)
Northern		Ref
North western		0.97(0.59, 1.61)
Southern		0.26***(0.16, 0.42)
Western		0.98(0.52, 1.82)

*p<0.05

**p<0.01

***p<0.001 Ref = Reference Category.

Source: Computed from the 2019 Sierra Leone Demographic and Health Survey.

Higher odds of quality ANC were noted among women covered by health insurance (aOR = 3.48; CI = 1.40, 8.64) compared to women who were not covered with. Women with Mende ethnic group were seen to have a higher odds of quality ANC (aOR = 2.58; CI = 1.79, 3.72) compared to women with Temne ethic group. The analysis further showed that women from Eastern region (aOR = 0.29, CI = 0.18, 0.46) and Southern region (aOR = 0.26, CI = 0.16, 0.42) were less likely to receive quality ANC in comparison with women from the Northern region.

## Discussion

The goal of reducing maternal and neonatal mortality is being prioritised globally and measures are being put in place by several countries to reduce this rate. We investigated the prevalence and characteristics of women who receive quality ANC in Sierra Leone. We found that socio-economic disadvantaged women, health insurance coverage, ethnicity, region, place of residence, frequency of listening to radio, and getting medical help for self: permission to go, had significant relationship with quality ANC utilisation.

In Sierra Leone, interventions such as FHCI have been implemented to increase patronage and quality of ANC services. The FHCI was found to favor the rich and the educated more than those on the lower end of the wealth quintile or with little or no education [24]. In accordance, this study found that poor socio-economic status remains a barrier to quality ANC. Women from most disadvantaged socio-economic backgrounds are less likely to have quality ANC compared to those from moderately socio-economic disadvantaged settings. This directly supports the findings of Obse & Ataguba [25] that socio-economically disadvantaged women are unable to utilize the recommended ANC services. Given that socio-economic disadvantage is attributable to low socio-economic status (SES), women in this category (SES) had lesser quality ANC comparatively. Women with higher SES are therefore more likely to seek quality care from facilities. Also, it is notable that an increase in wealth and educational level increases the quality of ANC [21]. This could be attributed to the argument of Obse & Ataguba [25] that the health care system in Sierra Leone may not be sensitive to the cultural needs of the poor and less educated women. As such, there is a need for policy makers to re-evaluate their policies to serve the needs of socio-economically disadvantaged women.

In certain parts of Africa (Western and Southern), it was found that women who are household heads have higher odds of going to the facility to seek healthcare [26]. This assertion corroborates the finding of our study that women with difficulty in getting permission to seek medical care for themselves had a lower likelihood of utilizing quality ANC. From the background characteristics, it was noted that a higher percentage (90.7%) of women that had no issue with permission to seek medical help had quality ANC compared to a lesser percentage with those that admitted difficulty in getting permission. It is however not surprising that, after controlling for other factors, the latter women have a higher likelihood of having quality ANC than those with no problem with seeking permission.

The place of residence is considered a determinant of ANC utilization. Tessema et. al. [4] and Shibre et al [27] asserted that women in rural settings tend to have a comparatively lower quality of ANC. This is however contrary to the finding in our studies. We found that women living in rural areas were more likely to utilise quality ANC services. This could be attributed to the FHCI which has helped in narrowing the wealth related inequality in MCH which includes ANC visits in Sierra Leone [15]. However, the existing residential inequality in may be due implementation gaps in the FCHI, considering that the FCHI was intended to overcome this gap. The government of Sierra Leone should review the FCHI and consider more measurable implementation indicators in order for the country to track the extent of implementation with ease. Besides, much synergistic effort is required between the Health Ministry and all maternal health stakeholders, whilst ensuring close monitoring and supervision at the facility level in order to ensure that all women receive quality ANC, irrespective of geographical location.

We also believe that the introduction of the National Health Sector Strategic Plan in 2017 by the Ministry of Health and Sanitation and the Diaspora Engagement project of the International Organisation for Migration in strengthening Sierra Leonean National Health Care Capacity have yielded positive results by making health care accessible, affordable and equitable in Sierra Leone [28]. Also, some studies showed that poorer women seem to have higher fertility rate and this can influence their increased demand for health services including ANC visits [29,30]. Given that most rural settings comparatively have higher proportion of poorer people [28], this could translate into the higher demand for quality ANC services in rural settings than urban ones.

We also found that women who listen to radio once a week have a higher chance of having quality ANC than those who do not listen at all. From previous studies, mass media especially radio serves as a source of information on health issues [31–33]. Benefo [32] and Lupton [33] argued that women receive information when needed through digital media. This shows that digital media plays a role in information dissemination and radio is one of such sources. This thus behooves the Ministry of Health and Sanitation of Sierra Leone to take advantage of the media to increase education on the importance of quality ANC utilization.

Even though other studies found distance to health facility as a barrier to quality ANC [1,13], the finding from our study was otherwise. Distance to health facility was not a barrier for women who were seeking health care for themselves. A plausible explanation is the introduction of the FCHI which eliminated medical fees and aided the provision of drugs and treatment at no cost. We believe this has lessened the financial barriers and enabled women to be able to access quality ANC services regardless of the distance. Since this service has helped in narrowing the wealth inequality [15].

We also found that women with health insurance coverage had a higher likelihood of utilizing quality ANC as compared to their counterparts with no health insurance coverage. This affirms the findings of Dadjo, Ahinkorah and Yaya [34] and Aboagye et al [35] that also found health insurance coverage has a significant association with the utilization of ANC. This could be attributed to the fact that health insurance eliminates the financial barriers that could have prevented women from seeking quality ANC services. We also observed that women from the Eastern and Southern region were less likely to utilize quality ANC as compared to women from the Northern region. Considering that the Eastern region has locations which happens to be the epicenters of the Ebola outbreak (e.g. Kailahun district) [36], it is possible that women’s motivation to access the health facility due to the fear deadly nosocomial infections, especially as pregnant women tend to have compromised immunity [37].

Effective community-based health education programs can be rolled out to educate women about the safety of health facilities and to remind them on the importance of quality ANC. Regarding Southern region, considering the limited health facilities, some authors have described it as surprising for women to have expected ANC [38]. Consequently, it is not surprising that women in the region had relatively lower odds of receiving quality ANC. Existing policies on maternal wellbeing need to priorities health promotion interventions, in order for women to appreciate that accessing quality ANC is extremely important for the wellbeing of themselves and their newborns.

### Strengths and limitations

The strength of the study is rooted in the robust analytical and statistical method used to enhance the trustworthiness of our findings. Besides, we provided detailed methodological procedure to enhance the replicability of this study. The use of nationally representative data also enables the generalizability of the study to all women in the reproductive age group in Sierra Leone. These notwithstanding, given that DHS employs a cross-sectional design, our study was not able to determine causality between the various factors. Since pre-existing data was used, it was not possible to include some indicators for measuring quality ANC, including but not limited to quantified distance and time spent to reach the health facility. Additionally, since the responses were self-reported, there is a possibility of recall bias, hence the interpretation of the findings should be done with some caution.

## Conclusion

The socio-economic disadvantaged status of women, place of residence as well as region, frequency of listening to radio, ethnicity, getting medical help for self: permission to go, and health insurance coverage were found to have a significant relationship with the utilization of quality ANC in Sierra Leone. Policy makers in the health sector of Sierra Leone need to consider measures to boost patronage of quality ANC in Sierra Leone by targeting the socio-economically disadvantaged women. Targeting these sub-groups with pro-MCH interventions such as encouraging education (either formally or informally) and creating job avenues would go a long way to help the socio-economically disadvantaged women. The policy makers should also consider re-evaluating their policies to be sensitive to the cultural needs of women to encourage socio-economic disadvantaged women to utilize quality ANC and MHC services.

## Abbreviations

ANC: Antenatal Care
AORs: Adjusted Odds Ratio
CEDAW: Convention on the Elimination of All Forms of Discrimination Against Women
CIs: Confidence Intervals
DHS: Demographic and Health Survey
FHCI: Free Health Care Initiative
ICESCR: International Covenant on Economic, Social and Cultural Rights
ICF: Inner-City Fund
LMICs: Low- And Middle-Income Countries
MMR: Maternal Mortality Rate
ORs: Odds Ratios
SDG: Sustainable Development Goals
SDG: Sustainable Development Goals
SES: Socio-Economic Status
SSA: Sub-Saharan Africa
WHO: World Health Organisation
